# The Cost of Pushing Pills: A New Estimate of Pharmaceutical Promotion Expenditures in the United States

**DOI:** 10.1371/journal.pmed.0050001

**Published:** 2008-01-01

**Authors:** Marc-André Gagnon, Joel Lexchin

In the late 1950s, the late Democratic Senator Estes Kefauver, Chairman of the United States Senate's Anti-Trust and Monopoly Subcommittee, put together the first extensive indictment against the business workings of the pharmaceutical industry. He laid three charges at the door of the industry: (1) Patents sustained predatory prices and excessive margins; (2) Costs and prices were extravagantly increased by large expenditures in marketing; and (3) Most of the industry's new products were no more effective than established drugs on the market [1]. Kefauver's indictment against a marketing-driven industry created a representation of the pharmaceutical industry far different than the one offered by the industry itself. As Froud and colleagues put it, the image of life-saving “researchers in white coats” was now contested by the one of greedy “reps in cars” [2]. The outcome of the struggle over the image of the industry is crucial because of its potential to influence the regulatory environment in which the industry operates.

Fifty years later, the debate still continues between these two depictions of the industry. The absence of reliable data on the industry's cost structures allows partisans on both sides of the debate to cite figures favorable to their own positions. The amount of money spent by pharmaceutical companies on promotion compared to the amount spent on research and development is at the heart of the debate, especially in the United States. A reliable estimate of the former is needed to bridge the divide between the industry's vision of research-driven, innovative, and life-saving pharmaceutical companies and the critics' portrayal of an industry based on marketing-driven profiteering.

IMS, a firm specializing in pharmaceutical market intelligence, is usually considered to be the authority for assessing pharmaceutical promotion expenditures. The US General Accounting Office, for example, refers to IMS numbers in concluding that “pharmaceutical companies spend more on research and development initiatives than on all drug promotional activities” [3]. Based on the data provided by IMS [4], the Pharmaceutical Research and Manufacturers of America (PhRMA), an American industrial lobby group for research-based pharmaceutical companies, also contends that pharmaceutical firms spend more on research and development (R&D) than on marketing: US$29.6 billion on R&D in 2004 in the US [5] as compared to US$27.7 billion for all promotional activities.[4]

In this paper, we make the case for the need for a new estimate of promotional expenditures. We then explain how we used proprietary databases to construct a revised estimate and finally, we compare our results with those from other data sources to argue in favor of changing the priorities of the industry.

## The Case for a New Estimate of Pharmaceutical Promotion

There are many concerns about the accuracy of the IMS data. First, IMS compiles its information through surveys of firms, creating the possibility that companies may systematically underestimate some of their promotional costs to enhance their public image. Second, IMS does not include the cost of meetings and talks sponsored by pharmaceutical companies featuring either doctors or sales representatives as speakers. The number of promotional meetings has increased dramatically in recent years, going from 120,000 in 1998 to 371,000 in 2004 [6]. In 2000, the top ten pharmaceutical companies were spending just under US$1.9 billion on 314,000 such events [7]. Third, IMS does not include the amount spent on phase IV “seeding” trials, trials designed to promote the prescription of new drugs rather than to generate scientific data. In 2004, 13.2% (US$4.9 billion) of R&D expenditures by American pharmaceutical firms was spent on phase IV trials [5]. Almost 75% of these trials are managed solely by the commercial, as opposed to the clinical, division of biopharmaceutical companies, strongly suggesting that the vast majority of these trials are done just for their promotional value [8].

Finally, IMS data seem inconsistent with estimates based on the information in the annual reports of pharmaceutical companies. For example, in an accounting study based on the annual reports of ten of the largest global pharmaceutical firms, Lauzon and Hasbani showed that between 1996 and 2005, these firms globally spent a total of US$739 billion on “marketing and administration.” In comparison, these same firms spent US$699 billion in manufacturing costs, US$288 billion in R&D, and had a net investment in property and equipment of US$43 billion, while receiving US$558 billion in profits [9].

Annual reports, however, have their own limitations. First, pharmaceutical firms are multinational and diversified; their annual reports provide no information on how much they spend on pharmaceutical marketing, as compared to the marketing of their non-pharmaceutical products, and they do not provide information about how much is spent on marketing specifically in the US. Second, annual reports merge the categories of “marketing” and “administration,” without delineating the relative importance of each. Finally, “marketing” is a category that includes more than just promotion; it also includes the costs of packaging and distribution. In terms of offering a more precise estimate of overall expenditures on pharmaceutical promotion in the US, annual reports are thus far from satisfactory.

In the absence of any collection of information on promotional spending by government or any other noncommercial source, the market research company IMS has long been the only source of such information, which it gains by surveying pharmaceutical firms. Since 2003, however, the market research company CAM has been providing comprehensive information on promotion expenditures by surveying doctors instead of firms. (In July 2005, CAM was merged into the Cegedim Group, another market research company.) We chose to compare IMS data to those produced by CAM in order to provide a more accurate estimate of promotional spending in the US. Other proprietary sources of data do not break down promotional expenditures into different categories and therefore were not used in our comparison.

## Methods

According to its Web site (http://www.imshealth.com/), IMS provides business intelligence and strategic consulting services for the pharmaceutical and health care industries. It is a global company established in more than 100 countries. IMS gathers data from 29,000 data suppliers at 225,000 supplier sites worldwide. It monitors 75% of prescription drug sales in over 100 countries, and 90% of US prescription drug sales. It tracks more than 1 million products from more than 3,000 active drug manufacturers. IMS data for 2004 were obtained from its Web site for the amount spent on: visits by sales representatives (detailing), samples, direct-to-consumer advertising, and journal advertising.

The Cegedim Web site (http://www.cegedim-crm.com/index.php?id=12) describes CAM as a global company dedicated to auditing promotional activities of the pharmaceutical industry, established in 36 countries worldwide. CAM annually surveys a representative sample of 2,000 primary care physicians and 4,800 specialists in a variety of specialties in selected locations in the US. From CAM's newsletter [10], we obtained access to data from CAM for the same promotion categories as from IMS. In addition, CAM provided figures for the amount of spending on company-sponsored meetings, e-promotion, mailings, and clinical trials.

We used 2004 as the comparison year because it was the latest year for which information was available from both organizations. We focused on the US because it is the only country for which information is available for all important promotional categories. The US is also, by far, the largest market for pharmaceuticals in the world, representing around 43% of global sales [11,12] and global promotion expenditures [10,13].

We asked both CAM and IMS about the procedures that they used to collect information on different aspects of promotion. Based on the answers we received, we determined the relevant figures for expenditures for samples and detailing. Each author independently decided on which values should be used, based on an understanding of the methods that the companies used to collect the information and the limitations of those methods. Differences were resolved by consensus.

We queried CAM and IMS about the estimated value of unmonitored promotional expenditures. IMS did not provide an answer to this question. In order to validate its estimates, CAM relies on a validation committee that includes representatives from various pharmaceutical firms, including Merck, Pfizer, Bristol-Myers Squibb, Eli Lilly, Aventis, Sanofi-Synthelabo, AstraZeneca, and Wyeth. Under a confidentiality agreement, the firms supply CAM with internal data related to their detailing activity and promotional costs in the US. Through the validation committee, CAM can thus compare totals obtained through its own audits with the firms' internal data about their promotional budgets in order to evaluate if all promotion has been properly audited through its physician surveys. As a result of this comparison, CAM's validation committee considers that about 30% of promotional spending is not accounted for in its figures. CAM is unable to provide an exact breakdown of unmonitored promotion, but it believes that around 10% is due to incomplete disclosure and omissions by surveyed physicians and the remaining 20% comes from a combination of promotion directed at categories of physicians that are not surveyed, unmonitored journals in which pharmaceutical promotion appears, and possibly unethical forms of promotion. We adjusted total expenditures to account for this unreported 30%.

## Results

For 2004, CAM reported total promotional spending in the US of US$33.5 billion [10], while IMS gave the figure of US$27.7 billion for the same year [4]. Both CAM and IMS cited the media intelligence company CMR as the source for the amount spent on direct-to-consumer advertising (US$4 billion), and they also gave the same figure for journal advertising (US$0.5 billion).

There were two major differences between the two sets of figures: the amounts spent on detailing and the amounts spent on samples. IMS estimated the amount spent on detailing at US$7.3 billion [4] versus US$20.4 billion for CAM [10], and while IMS gave a retail value of US$15.9 billion for samples [14], CAM estimated a wholesale value of US$6.3 billion [10].

Using the IMS figure of US$15.9 billion for the retail value of samples, and adding the CAM figures for detailing and other marketing expenses after correcting for the 30% estimate of unaccounted promotion, we arrived at US$57.5 billion for the total amount spent in the US in 2004, more than twice what IMS reported (see Table 1).

**Table 1:**
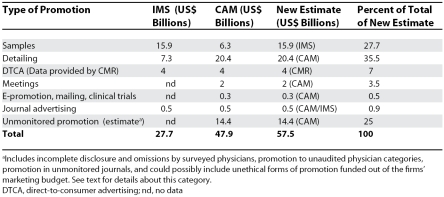
Pharmaceutical Marketing Expenditures in the United States in 2004: Data from IMS, CAM, and Our New Estimate

## Discussion

Our revised estimate for promotional spending in the US is more than twice that from IMS. This number compares to US$31.5 billion for domestic industrial pharmaceutical R&D (including public funds for industrial R&D) in 2004 as reported by the National Science Foundation [15].

However, even our revised figure is likely to be incomplete. There are other avenues for promotion that would not be captured by either IMS or CAM, such as ghostwriting [16] and illegal off-label promotion [17]. Furthermore, items with promotional potential such as “seeding trials” or educational grants might be included in other budgets and would not be seen in the confidential material provided to CAM's validation committee.

IMS and CAM data were used for comparison purposes for a number of reasons: data from both were publicly available, both operate on a global scale and are well regarded by the pharmaceutical industry, both break down their information by different categories of promotion, and, most importantly, they use different methods for gathering their data, thereby allowing us to triangulate on a more accurate figure for each category.

Methodological differences between the ways that IMS and CAM collect data will affect the values for promotional spending depending on the category being considered. Because of the problematic nature of some data from each firm, we believe that the most precise picture of industry spending can be obtained by selectively using both sets of figures.

CAM compiles its data on the value of detailing and samples through systematic surveys of primary care providers and specialists and by estimating an average cost for each visit by a sales representative according to the type of physician. By contrast, IMS compiles its data on the value of detailing through surveys of firms, while its data on samples are obtained by monitoring products directly from manufacturers.

There is a significant discrepancy between the two sets of data in the cost of detailing: US$7.3 billion for IMS and US$20.4 billion for CAM. This difference can be explained by the fact that CAM offers a more complete data set since it includes in the average cost of a call (a sales representative's visit to a physician) not only the “cost to field the rep” (salary and benefits of the representative and the transportation cost) but also the costs for the area and regional managers, the cost of the training, and the cost of detail aids such as brochures and advertising material. By contrast, in reporting the cost of detailing IMS only considers the “cost to field the rep.” Furthermore, relying on physician-generated data to estimate the amount spent on detailing is likely to give a more accurate figure than using figures generated by surveying firms. Companies may not report some types of detailing, for example, the use of sales representatives for illegal off-label promotion, whereas doctors are not likely to distinguish between on- and off-label promotion and would report all encounters with sales representatives.

In the case of samples, there is also a large difference between the IMS (US$15.9 billion) and CAM (US$6.3 billion) estimates. CAM estimates the amount spent on samples by multiplying the number of samples declared by physicians with their wholesale value. The latter is determined by using the average wholesale price (AWP), which is the amount set by manufacturers and used by Medicare in the US to determine reimbursement. CAM then divides that amount in half to account for the fact that samples are frequently given out in small dosage forms. CAM admits, however, that the amount for samples is understated because, when physicians fill out their survey, any quantity of samples of the same product left during a call is considered to be only one sample unit. CAM's calculations also rely on the AWP, which has been criticized for not taking into account the various discounts and rebates that are negotiated between manufacturers and purchasers [18].

IMS provides exact figures for the retail value for samples by monitoring 90% of all pharmaceutical transactions and by tracking products directly from manufacturers. This method for calculating the value of samples is much more direct than CAM's and therefore is likely to be subject to less error.

Using the wholesale value for samples, the CAM figure would be appropriate if we were arguing that the money spent on samples should go to another activity such as R&D. However, we have used the retail value of samples because this is consistent with companies' reporting of drugs they donate [19]. As these are both categories of products that are being distributed without a charge to the user, it is inconsistent for donations to be reported in terms of retail value and samples in terms of wholesale value.

We believe that it is appropriate to correct for unmonitored promotion and that the figure we used is a reliable estimate. The 30% correction factor is based on a direct comparison that CAM is able to make between the data it collects through its surveys and the amount reported by companies.

There are other ways of combining the data that we have presented, but with the exception of choosing the lower amounts for detailing and samples and ignoring the 30% for unmonitored promotion, all of them yield a higher figure than the one from IMS. Some examples of alternative estimates follow: using the CAM estimate for the wholesale value of samples and the 30% adjustment, the total amount would be US$47.9 billion; without the 30% adjustment CAM's estimate is US$33.5 billion. Adding the figures for the categories that IMS does not cover (meetings, e-promotion, mailing, clinical trials) boosts its estimate to US$31 billion; using the lower figures for detailing and samples plus the CAM amounts for the other categories and applying the 30% adjustment gives an amount of US$29.1 billion. Therefore, the actual amount could range from a low of US$27.7 billion to a high of US$57.5 billion. Our analysis shows, however, that the figure of US$57.5 billion is the most appropriate one when using the most relevant figures for each category of promotional spending.

Excluding direct-to-consumer advertising, CAM considers that around 80% of the remaining promotion is directed towards physicians, with 20% of this figure going to pharmacists. (IMS does not provide any comparable values.) With about 700,000 practicing physicians in the US in 2004 [20], we estimate that with a total expenditure of US$57.5 billion, the industry spent around US$61,000 in promotion per physician. As a percentage of US domestic sales of US$235.4 billion [21], promotion consumes 24.4% of the sales dollar versus 13.4% for R&D.

Our new estimate of total promotion costs and promotion as a percentage of sales is broadly in line with estimates of promotional or marketing spending from other sources. The annual reports of Novartis distinguish “marketing” from “administration.” Marcia Angell extrapolates from this annual report to the entire industry and calculates a figure of US$54 billion spent on pharmaceutical promotion in the US in 2001 [22]. As a proportion of sales, she estimates 33% is spent on marketing. Using similar methodology, the Office of Technology Assessment derived an estimate for marketing costs in the US by extrapolating from the cost structure of Eli Lilly. The Office of Technology Assessment considers that firms spend around 22.5% of their sales on marketing [23]. Based on United Nations Industrial Development Organization estimates, a report from the Organization for Economic Cooperation and Development estimated that, in 1989, pharmaceutical firms globally spent 24% of their sales on marketing [24], but few details of the methodology used were provided, making it impossible to verify the accuracy of the estimate. Finally, in 2006 Consumers International surveyed 20 European pharmaceutical firms to obtain more information about their exact expenditures on drug promotion. Among the 20 firms contacted, only five agreed to provide separate figures for marketing, which ranged from 31% to 50% of sales depending on the firm [25].

The results are also consistent with data on the share of revenue allocated to “marketing and administration” according to annual reports of large pharmaceutical companies, if we consider that the largest part of “marketing and administration” is devoted to promotion. Lauzon and Hasbani found that 33.1% of revenues was allocated to “marketing and administration” [9], similar to the 31% reported by the Centers for Medicare and Medicaid Services [26] and the 27% from Families USA [27].

The value of our estimate over these others is that it is not based on extrapolating from annual reports of firms that are both diversified and multinational. Our estimate is driven by quantifiable data from highly reliable sources and concerns only the promotion of pharmaceutical products in the US. The derivation of our figure is thus transparent and can form the basis for a vigorous debate.

## Conclusion

From this new estimate, it appears that pharmaceutical companies spend almost twice as much on promotion as they do on R&D. These numbers clearly show how promotion predominates over R&D in the pharmaceutical industry, contrary to the industry's claim. While the amount spent on promotion is not in itself a confirmation of Kefauver's depiction of the pharmaceutical industry, it confirms the public image of a marketing-driven industry and provides an important argument to petition in favor of transforming the workings of the industry in the direction of more research and less promotion.

## Supporting Information

Abstract S1 English abstract (20 KB DOC).

Alternative Language Abstract S1 Translation of the abstract into French by MAG (20 KB DOC).

Appendix S1 The Web links displayed in the footnotes 4,7,10,13,14, and 21 are now defunct. In order to make accessible all data we used for this article, this appendix provides supporting information or alternative sources for the defunct Web links. (994 KB PDF).

## Abbreviations

AWP: average wholesale price
PhRMA: Pharmaceutical Research and Manufacturers of America
R&D: research and development

## References

[pmed-0050001-b001] Kefauver E (1965). In a few hands.

[pmed-0050001-b002] Froud J, Johal S, Leaver A, Williams K (2006). Financialization and strategy: narrative and numbers.

[pmed-0050001-b003] General Accounting Office (2002). Prescription drugs: FDA oversight of direct-to-consumer advertising has limitations. http://www.gao.gov/new.items/d03177.pdf.

[pmed-0050001-b004] IMS Health (2005). Total U.S. promotional spend by type, 2004. http://www.imshealth.com/ims/portal/front/articleC/0,2777,6599_49695992_75406357,00.html.

[pmed-0050001-b005] Pharmaceutical Research and Manufacturers of America (2006). Pharmaceutical industry profile 2006. http://www.phrma.org/files/2006%20Industry%20Profile.pdf.

[pmed-0050001-b006] Hensley S, Martinez B (2005 July 15). To sell their drugs, companies increasingly rely on doctors. Wall Street Journal.

[pmed-0050001-b007] Quintiles Transnational (2001). Promoting drugs through physician meetings and events: Pfizer leads the way; antidepressants are top category. http://www.quintiles.com/products_and_services/informatics/scott_levin/press_releases/press_release/1,1254,209,00.html.

[pmed-0050001-b008] La Puma J, Seltzer J (2002). Phase IV market steams ahead. CenterWatch.

[pmed-0050001-b009] Lauzon L-P, Hasbani M (2006). Analyse socio-économique: industrie pharmaceutique mondiale pour la période de dix ans 1996–2005.

[pmed-0050001-b010] CAM Group (2005). Total U.S. promotional activity for 2004. CAM USA Newsletter. http://csd.cam-group.com/www_assets/pages/downloads/USNewsletterQ404.pdf.

[pmed-0050001-b011] IMS Health (2006). Channel distribution by U.S. sales. http://www.imshealth.com/ims/portal/front/articleC/0,2777,6652_80408863_80411875,00.htm.

[pmed-0050001-b012] IMS Health (2006). Global pharmaceutical sales, 1999–2006. http://www.imshealth.com/ims/portal/front/articleC/0,2777,6652_80528184_80528202,00.html.

[pmed-0050001-b013] CAM Group (2005). 4th quarter 2004 promotional activity. CAM Group News. http://csd.cam-group.com/www_assets/pages/downloads/CAMNewsletterQ404.pdf.

[pmed-0050001-b014] IMS Health (2004). Total U.S. value of free product samples, 2004. http://www.imshealth.com/ims/portal/front/articleC/0,2777,6599_78152267_78152297,00.html.

[pmed-0050001-b015] National Science Foundation (2006). U.S. industrial R&D performers report increased expenditures for 2004. http://www.nsf.gov/statistics/infbrief/nsf07304/#notes.

[pmed-0050001-b016] Gøtzsche PC, Hróbjartsson A, Johansen HK, Haahr MT, Altman DG (2007). Ghost authorship in industry-initiated randomised trials. PLoS Med.

[pmed-0050001-b017] Steinman MA, Bero LA, Chren MM, Landefeld CS (2006). Narrative review: the promotion of gabapentin: an analysis of internal industry documents. Ann Intern Med.

[pmed-0050001-b018] Gencarelli DM (2005). One pill, many prices: variation in prescription drug prices in selected government programs. National Health Policy Forum. http://www.nhpf.org/pdfs_ib/IB807_DrugPricing_08-29-05.pdf.

[pmed-0050001-b019] Pharmaceutical Research and Manufacturers of America (2005). America's pharmaceutical companies increase Katrina donations; more than $100 million in medicines, cash donated so far. http://www.liberty-page.com/issues/comparison/pharm.html.

[pmed-0050001-b020] Organization for Economic Cooperation and Development (2006). OECD health data 2006. Organization for Economic Cooperation and Development.

[pmed-0050001-b021] IMS Health (2004). U.S. purchase activity by channel, 2004. http://www.imshealth.com/ims/portal/front/articleC/0,2777,6599_49695983_69891354,00.html.

[pmed-0050001-b022] Angell M (2004). The truth about the drug companies: how they deceive us and what to do about it.

[pmed-0050001-b023] Office of Technology Assessment (1993). Pharmaceutical R&D: costs, risks and rewards.

[pmed-0050001-b024] Jacobzone S (2000). Pharmaceutical policies in OECD countries: reconciling social and industrial goals.

[pmed-0050001-b025] Consumers International (2006). Branding the cure: a consumer perspective on corporate social responsibility, drug promotion and the pharmaceutical industry in Europe.

[pmed-0050001-b026] Centers for Medicare and Medicaid Services (2003). Health care industry market update: pharmaceuticals. http://www.fdcpa.com/CMS%20Pharmaceuticals%20Jan%202003.pdf.

[pmed-0050001-b027] Families USA (2002). Profiting from pain: where prescription drug dollars go. http://www.familiesusa.org/assets/pdfs/PPreport89a5.pdf.

