# Supplementary material for: The Cost of Pushing Pills: A New Estimate of Pharmaceutical Promotion Expenditures in the United States
Source: PLoS Med. 2008 Jan 1;5(1):e1. doi: 10.1371/journal.pmed.0050001 (PMC2174966; doi:10.1371/journal.pmed.0050001)
Supplement: Supplementary file 1 [file pmed.0050001.sd003.pdf]

The Cost of Pushing Pills: A New Estimate of Pharmaceutical Promotion Expenditures in the United States

APPENDIX

The web links displayed in the footnotes 4,7,10,13,14 and 21 are now defunct. In order to make accessible all data we used for this article, this appendix provides supporting information or alternative sources for the defunct web links.

Footnote 4:

The data the footnote refers to are also available at the Kaiser Family Foundation (2006). Trends and Indicators in the Changing Health Care Marketplace. Accessed December 3 2007 at http://www.kff.org/insurance/7031/ti2004-1-20.cfm.

Footnote 7:

The PDF document the web link refers to is enclosed on the next pages.

Quintiles Transnational (2001) Promoting drugs through physician meetings and events: Pfizer leads the way; antidepressants are top category. Accessed 3 March 2002 at http://www.quintiles.com/products_and_services/informatics/scott_levin/press_releases/press_release/1,1254,209,00.html.

Press Releases

FOR IMMEDIATE RELEASE

Printer-friendly Version

Promoting Drugs Through Physician Meetings and Events: Pfizer Leads the Way; Antidepressants Are Top Category

March 26, 2001

NEWTOWN, Pa. (March 26, 2001) -- Pharmaceutical meetings and events continue to grow as integral elements in overall promotion of prescription drugs.

According to Scott-Levin's ***Physician Meeting & Event Audit***, a survey of more than 3,400 doctors, pharmaceutical companies spent $1.9 billion on events for physicians in 2000, 14% more than in 1999.

By comparison, the industry spent the same amount on direct-to-consumer ads for prescription products between January and September 2000, reports Scott-Levin's ***Direct-to-Consumer Advertising Audit*** and Competitive Media Reporting.
Pharmaceutical companies devoted an average of 17% of their promotional budgets to meetings and events, about the same proportion as in 1999. Total promotional spending for prescription drugs rose from $9 billion to $10.4 billion in 2000.
Scott-Levin projects that 314,022 physician events were held in 2000, an increase of about 11%. Event activity almost doubled between 1996 and 2000.

Top 10 Companies in 2000 Event Spending

Company	Event Spending (in millions)	% Change
Pfizer	$211.7	-12%
GlaxoSmithKline	$195.0	45%
Merck	$179.2	20%
Bristol-Myers Squibb	$106.3	9%
Eli Lilly	$99.9	45%
AstraZeneca	$84.6	9%
Pharmacia	$81.0	10%
Johnson & Johnson	$74.8	-2%
Aventis	$69.5	-6%
American Home Products	$62.0	25%
**Total Industry**	**$1,887.7**	**14%**

Top 5 Classes in 2000 Events

Class	Events	% Change
SSRI/SNRI antidepressants	22,838	31%
Cholesterol-reducing statins	16,560	18%
COX-2 inhibitors	13,815	22%
Antihistamines	10,395	25%
Proton pump inhibitors	9,624	71%
**Total Industry**	**314,022**	**11%**

Source: Scott-Levin's ***Physician Meeting & Event Audit***

For more information, please contact Jim Dietz, jim.dietz@scottlevin.com; Debbie Alcott, debbie.alcott@scottlevin.com; Nancy Robertone, nancy.robertone@scottlevin.com; or Dave Johnson, dave.johnson@scottlevin.com. All can be reached at (215) 860-0440; fax: (215) 860-5477.

Scott-Levin provides consulting services to more than 100 U.S. and international pharmaceutical clients. These services monitor key areas such as product promotion, industry trends, retail pharmacy activity, market performance, managed care and government affairs.
Scott-Levin's market research audits and strategic studies cover detailing, meetings and events, direct-to-consumer advertising, journal advertising, and other media. Taken together, Scott-Levin products can give clients the most accurate possible picture of total pharmaceutical promotion.
Scott-Levin is part of Quintiles Transnational, the world's leading provider of information, technology and services to bring new medicines to patients faster and improve health care. Headquartered near Research Triangle Park, N.C., Quintiles employs a global workforce of 18,000 in 39 countries. Quintiles is a member of the S&P 500 and Fortune 1000. For more information, visit the company's Web site at www.quintiles.com.

Copyright © 1998-2002 Quintiles Transnational Corp. or its subsidiaries. All rights reserved.

Footnote 10:

A scan of the PDF document the web link refers to is enclosed on next page.

CAM Group (2005) Total U.S. promotional activity for 2004. CAM USA Newsletter.

Accessed 22 July 2007 at http://csd.cam-

group.com/www_assets/pages/downloads/USNewsletterQ404.pdf.

CAM GROUP News

March, 2005

Monitoring Pharmaceutical Industry Promotion

Welcome to the March 2005 Edition of the CAM USA Newsletter!

Total U.S. Promotional Activity for 2004

**Promotional Channel Spending in the U.S.
Calendar Year 2004**

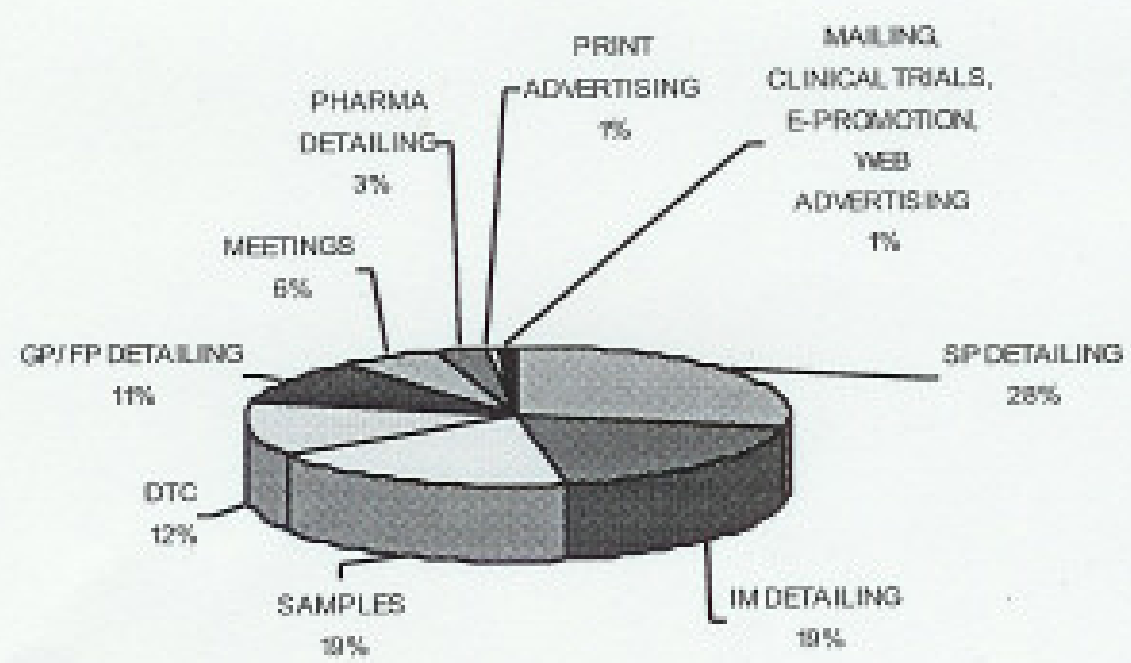

**Growth Across Media Channels
for the Calendar Year 2004**

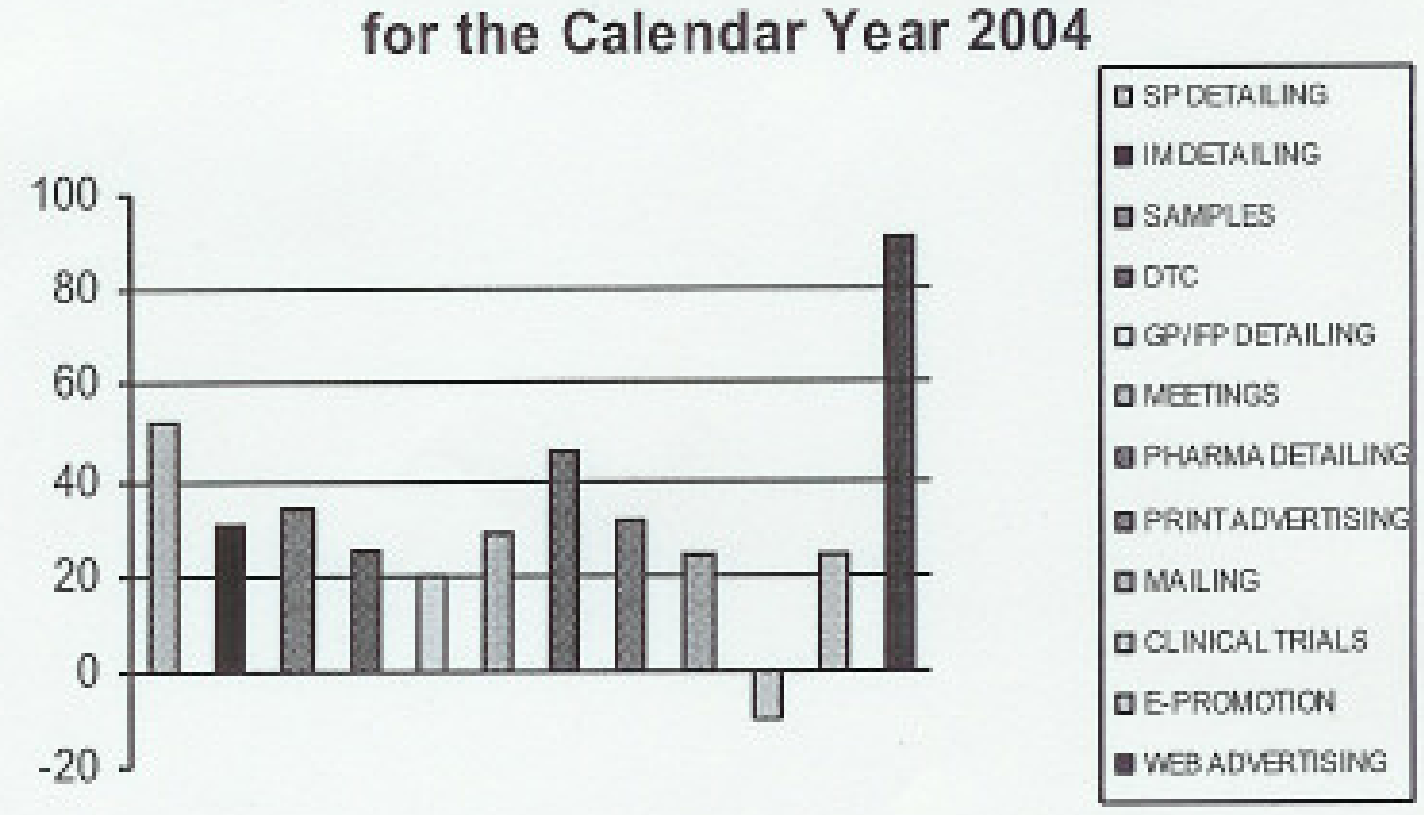

**DTC information provided by CMR*

Total promotional spending in the United States for 2004 reached $33.5 billion, ($8.2 billion for the latest quarter) as projected by CAM's promotional Spending Audit. This is a 34.6% increase in total promotional spending from the previous year. There were no significant changes in the allocation of spending among media channels from 2003 to 2004. Detailing was the leading channel for spend for the fourth quarter of 2004 and for the year. Detail spending amounted to $5 billion for Q4/2004 and it reached $20.5 billion, (approximately 61% of spending) for the year.

Web Advertising had the greatest growth in spending for 2004, up 91% from the previous year to reach $2.3 million. The majority of media channels had increases in promotional efforts from the previous year. Other increases in promotional activity include: Specialty Detailing (+52%), Sampling (+34%), DTC advertising (+25%), Journal advertising (+32%), and Mailing (+25%). The only channel to show a decrease in promotional spending activity is Clinical Trials (-10%).

Leading Therapeutic Areas

Q4/2004		2004	
Anti-hyperlipidemics	$783 mn	Antidepressants	$3.0 bn
Antidepressants	$729	Antihyperlipidemics	$2.8
Anti-infectives	$519	Anti-arthritics	$2.1
Anti-arthritics	$494	Anti-infectives	$2.0
Renin Angiotensin Antagonists	$454	Anti-ulcerants	$516

The antidepressant class remained the leading therapeutic area in promotional spending for the second consecutive year. Spending for this therapeutic class totaled $3.0 bn for 2004. Lexapro, reaching over $638 mn, followed by Zoloft ($478 mn) and Strattera ($376mn) helped lead the antidepressant therapeutic class to the number one spot for promotional spend for the full year 2004. Following the antidepressant therapeutic class was anti-hyperlipidemics ($2.8bn), and anti-arthritics ($2.1 bn). Taking a look at Q4/2004, promotional spending on anti-hyperlipidemics exceeded all other therapeutic areas to take the lead at $783 mn. Products in this therapeutic class that contributed to the strong promotional spend for Q4/2004 were lead by Crestor ($175mn), Vytorin ($154mn), and Lipitor ($111mn). The antidepressant therapeutic class came in second for promotional spend for Q4/2004, followed by the anti-infectives.

Footnote 13:

A scan of the PDF document the web link refers to is enclosed on next page.

CAM Group (2005) 4th quarter 2004 promotional activity. CAM Group News. Accessed 22 July 2007 at http://csd.cam-group.com/www_assets/pages/downloads/CAMNewsletterQ404.pdf.

CAM GROUP News
MONITORING PHARMACEUTICAL INDUSTRY PROMOTION WORLDWIDE
25 March 2005 – Volume 4 Issue 4

4th Quarter 2004 Promotional Activity

Worldwide spending reached $19.2 bn during the fourth quarter of 2004, and pushed total spending to $77.7 bn for all of 2004, as projected by CAM's Promotional Spending Audit.

Total Detailing Spend, which now splits out detailing to Nurses (in applicable markets) amounted to $54.3 bn, or slightly less than 70% of total 2004 spending. **Samples**, ranking second for both the quarter and MAT, reported spend of $2.1 and $8.7 billion, respectively.

North America led regional spending for the year, with $34.5 bn, followed by **Total Europe** with $19.9 bn, **Asia Pacific** with $19.3 bn and **Latin America** with $3.0 bn. **Global HQ** spending brought another $0.9 bn to the total.

2004

Mergers

2004 was an especially active year for the pharmaceutical industry. During the first quarter of 2004, **Yamanouchi** and **Fujisawa** announced a $7.5 billion merger, aimed at boosting their competitiveness against their rivals in the US and Europe. Yamanouchi's 2004 worldwide spend of $540 million (#22) added to Fujisawa's $280 million for the same time period (#46), will lift the new company, **Astellas Pharma** (effective in CAM Q2/05), into the Top 20 on a little more than 1% of total spending. In Japan the company will rank 7th in promotional spend ($0.6 bn).

In one of the largest mergers to take place in recent years, the announcement was made of **Sanofi-Synthelabo's** $65 billion acquisition of **Aventis**. The combined company, **Sanofi-Aventis** (effective in CAM Q1/05 with retroactive changes for 2004), accounts for $3.6 bn of total promotional spending and places 4th among all corporations. (**Aventis** formerly ranked 7th on $2.6 bn in spending; **Sanofi-Synthelabo**, 17th with $0.9 bn in spending.)

Already for 2005, Japanese companies **Daiichi** ($0.3 bn, 36th worldwide; $0.3 bn, 23rd in Japan) and **Sankyo** ($1.2 bn, 15th worldwide; $0.7 bn, 2nd in Japan) have confirmed the consolidation of their businesses, in a stock deal valued at $7.7 bn. This merger was followed a couple of weeks later by news that Belgian industrial group **Solvay** acquired one of France's leading pharmaceutical companies, **Fournier Pharma**, in a $1.7 billion deal. The combined company will rank 22nd with nearly $0.6 bn in promotional spend (Worldwide, Solvay presently ranks 24th, Fournier 94th).

WORLDWIDE PROMOTIONAL SPENDING
Corporate Mergers 2005
Current Spend and SOV
MAT/Q4/2004 – Total Spending in Constant Dollars

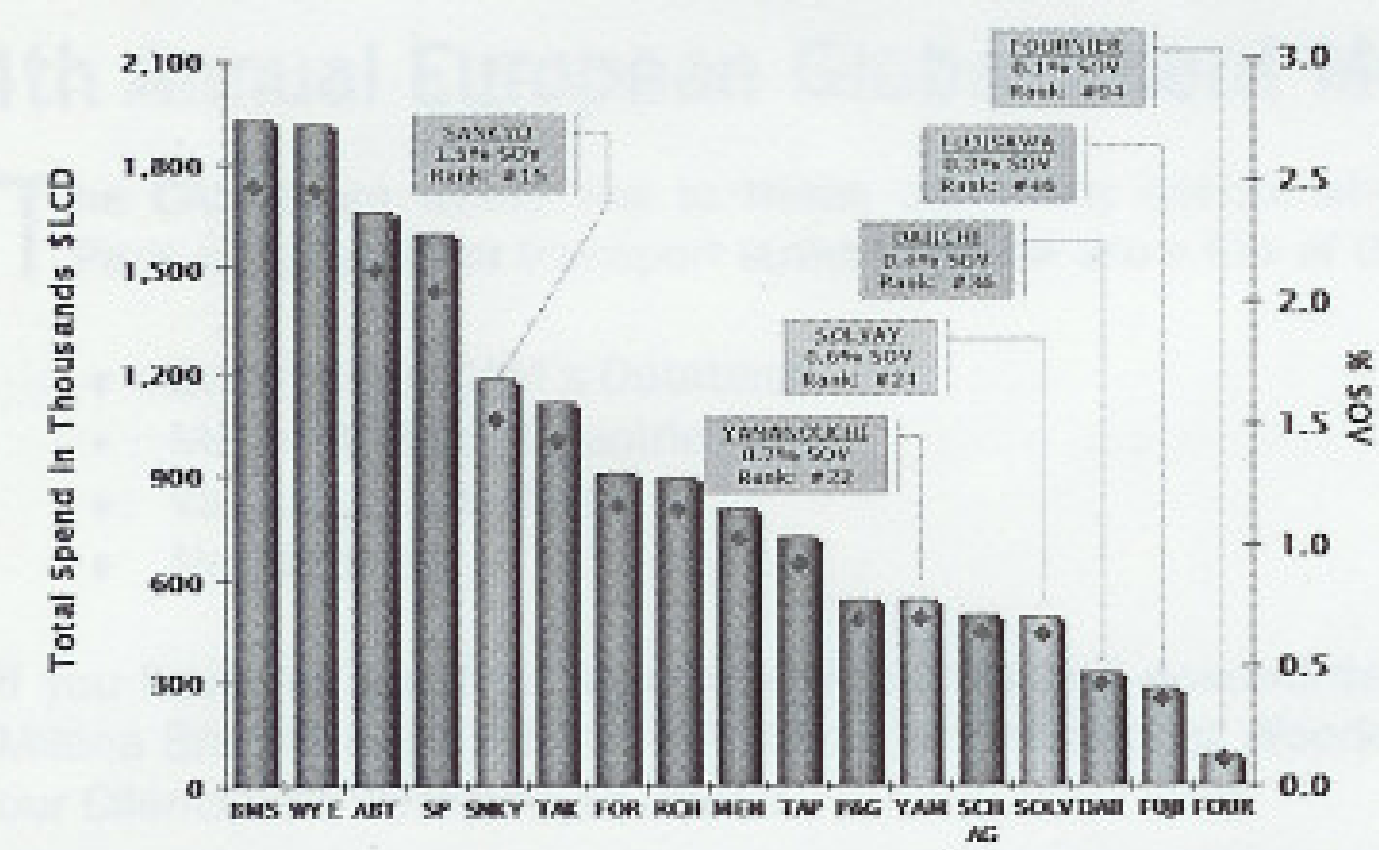

WORLDWIDE PROMOTIONAL SPENDING
Corporate Mergers 2005
Spend and SOV – Post Merger
MAT/Q4/2004 – Total Spending in Constant Dollars

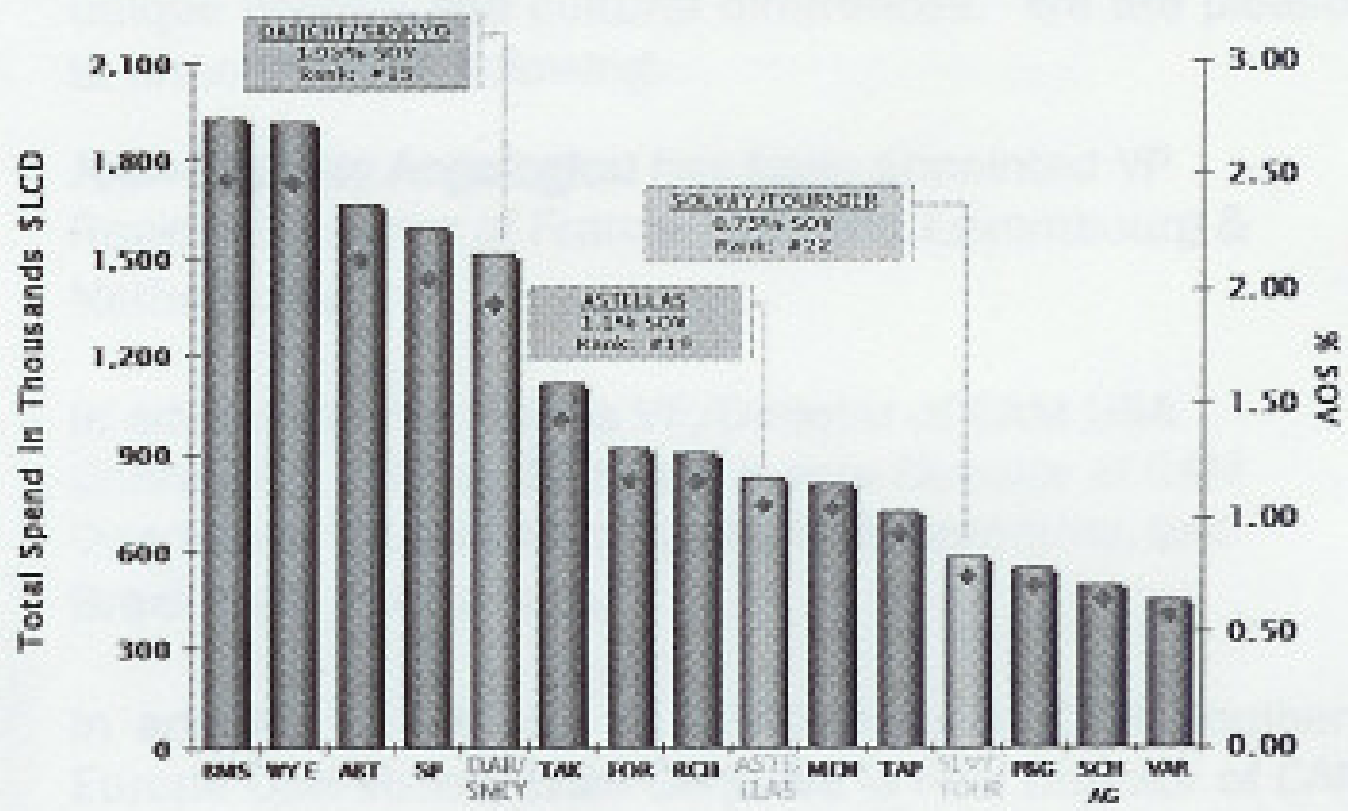

This acquisition serves to increase **Solvay's** activities in the pharmaceutical arena and enables them to capitalize on the success of **Fournier's** cardiovascular portfolio, in particular the cholesterol-lowering product **Lipanthyl** (sold as **TriCor** and **Abbott** in the USA). Solvay's corporate website reported this "transaction would expand Solvay Pharmaceuticals' business by more than one third in terms of sales." In terms of promotional efforts, Solvay concentrates primarily in the Cardiovascular (28%), Genito-Urinary (24%) and CNS (19%) therapy areas; Fournier is largely focused in Cardiovascular (60%), followed by Anti-Infectives and Genito-Urinary (13% each). Combined, the merged company would rise to 21st, 1.3% SOV, among companies promoting **Cardiovascular** (ATC1 **C**) products., (Separately, **Solvay** ranks 24th ($139.6 mn) in Cardiovascular spending; **Fournier**, 39th ($52.3 mn.)

Promotion

In a backlash from **Vioxx's** recall, promotional practices for pharmaceuticals continue to come under fire. **DTC** has become a favorite target for the very reason companies use it—wide reaching, high visibility — accounting $6.1 billion, or nearly 8% of total worldwide spending. The FDA panel reviewing Cox-2's safety, have called these consumer ads 'inappropriate'. As the **Vioxx** lawsuits come to trial, promotional practices will remain in the spotlight for changes to be made. Already some recent analyst reports foresee toned down, more subtle DTC ads for the future. In perhaps an effort to counter anti-Pharma sentiment fuelled by **Vioxx's** recall, the US Industry group, **PhMRA** have two publications on their website concerning promotional practices and guidelines for the pharmaceutical industry.

European drug companies walk a line between standing out against aggressive, US-style, DTC advertising and providing greater information to the public on medicines. DTC spending in Europe has remained somewhat steady where it accounts for 10% of the region's spending, or slightly more than $2.0 billion for the MAT/Q4/2004.

Footnote 14:

The data the footnote refers to are also available at the Kaiser Family Foundation (2006). Trends and Indicators in the Changing Health Care Marketplace. Accessed December 3 2007 at http://www.kff.org/insurance/7031/ti2004-1-20.cfm.

Footnote 21:

The data the footnote refers to are also available in the article

IMS Reports 8.3 Percent Dollar Growth in 2004 U.S. Prescription Sales (2005). Business Wire. Feb 14.
