# Supplementary material for: The Cost of Pushing Pills: A New Estimate of Pharmaceutical Promotion Expenditures in the United States
Source: PLoS Med. 2008 Jan 1;5(1):e1. doi: 10.1371/journal.pmed.0050001 (PMC2174966; doi:10.1371/journal.pmed.0050001)
Supplement: Supplementary file 2 [file pmed.0050001.sd001.doc]

**Abstract**

**Introduction**

The amount spent on pharmaceutical promotion versus research and development is central to the debate around whether the pharmaceutical industry is primarily driven by innovation or marketing. Information on promotional expenditures from IMS, the most widely quoted authority that surveys pharmaceutical firms, may not be reliable.

**Methods**

We accessed data from CAM, a global market research company dedicated to survey physicians to audit promotional and marketing activities undertaken by the pharmaceutical industry, compared its estimates to those from IMS and determined the relevant figures from both organizations in each category of promotional expenditures to arrive at a new estimate of total spending.

**Results**

Pharmaceutical promotion in the United States in 2004 is as high as $57.5 billion compared to the figure of $27.7 billion given by IMS. Excluding direct-to-consumers advertising and promotion towards pharmacists, the industry spent around $61,000 in promotion per practicing physician.

**Comment**

As a percent of U.S. domestic sales of $ 235.4 billion, promotion consumes 24.4% of the sales dollar versus 13.4% for R&D. In light of these numbers, one can safely argue that marketing is predominant over R&D in the pharmaceutical industry, contrary to the industry’s claim.
