# Supplementary material for: The Cost of Pushing Pills: A New Estimate of Pharmaceutical Promotion Expenditures in the United States
Source: PLoS Med. 2008 Jan 1;5(1):e1. doi: 10.1371/journal.pmed.0050001 (PMC2174966; doi:10.1371/journal.pmed.0050001)
Supplement: Supplementary file 3 [file pmed.0050001.sd002.doc]

**Translation of the abstract into French by Marc-André Gagnon**

**Résumé**

**Introduction**

La comparaison des dépenses par l’industrie pharmaceutique pour la promotion de ses produits par rapport aux dépenses en recherche et développement est au centre du débat pour déterminer si l’industrie pharmaceutique repose avant tout sur l’innovation ou le marketing. La source normalement citée pour déterminer les dépenses de promotion pharmaceutiques, IMS, n’est pourtant pas fiable.

**Méthode**

Nous avons accédé aux données colligées par CAM, une compagnie globale d’étude de marché qui, par des sondages auprès des médecins, détermine les dépenses de marketing et de promotion par l’industrie pharmaceutique. Nous avons comparé ces chiffres avec ceux d’IMS et, pour chaque catégorie de dépenses promotionnelles, nous avons déterminé les estimations les plus pertinentes pour chaque organisation afin de déterminer un nouveau montant de dépenses totales.

**Résultats**

La promotion pharmaceutique aux États-Unis en 2004 est ainsi estimée à $57.5 milliards, comparativement à l’estimation normalement citée d’IMS de $27.7 milliards. En excluant la publicité visant les consommateurs et les pharmaciens, l’industrie a dépensé environ $61,000 par médecin pratiquant.

**Commentaire**

Proportionnellement aux ventes domestiques aux États-Unis de $235 milliards, la promotion des produits pharmaceutiques représente donc 24.4% du chiffre d’affaires de l’industrie, comparativement à 13.4% pour la recherche et développement. À la lumière de ces chiffres, on peut prétendre sans crainte de se tromper que, dans l’industrie pharmaceutique actuelle, la promotion et le marketing joue une part beaucoup plus importante que la recherche et développement, contrairement à ce que prétend l’industrie.
